# Atrial fibrillation-related cardiomyopathy: a case report

**DOI:** 10.1186/1752-1947-1-111

**Published:** 2007-10-22

**Authors:** Simon TC Peake, Paresh A Mehta, Simon W Dubrey

**Affiliations:** 1 Department of Cardiology, The Hillingdon Hospital, Middlesex UK

## Abstract

Sustained chronic tachyarrhythmias often cause a deterioration of cardiac function known as tachycardia-induced cardiomyopathy or tachycardiomyopathy.

The incidence of tachycardia-induced cardiomyopathy is unknown, but in selected studies of patients with atrial fibrillation, approximately 25% to 50% of those with left ventricular dysfunction had some degree of tachycardia-induced cardiomyopathy. It is an important clinical entity due to the high incidence and potential reversibility of the disease process.

This case describes a cardiomyopathy induced by excess caffeine consumption. Six months following withdrawal of caffeine from the subject's diet, full resolution of symptoms occurred.

## Case History

**Figure 1:**
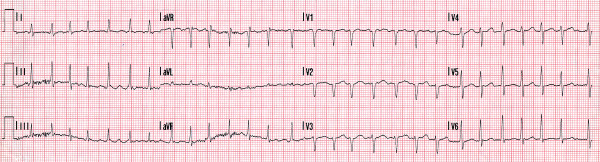
Resting electrocardiogram showing fast atrial fibrillation.

A 58 year-old male was admitted with a history of worsening dyspnoea and palpitations. He was previously fit and well. Physical examination was unremarkable aside from a fast irregular pulse. Thyroid function tests were normal. An electrocardiogram revealed atrial fibrillation with a ventricular rate of 169 beats per minute (Figure 1). Trans-thoracic echocardiography revealed a dilated left ventricle with a diastolic diameter of 6.2 cm (normal range: 3.9 – 5.6 cm), systolic diameter of 5.3 cm (2.0 – 3.8 cm) and a reduced ejection fraction of 45% (>70%). A diagnosis of dilated cardiomyopathy was made. The patient was commenced on digoxin, ramipril and warfarin. Coronary angiography showed normal coronary arteries and confirmed the global ejection fraction as 45%.

**Figure 2:**
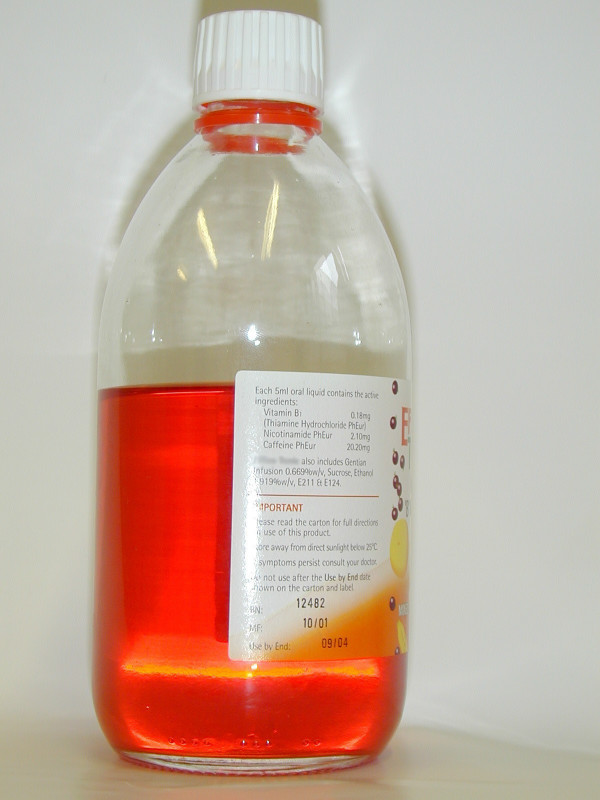
The stimulant beverage containing 4.04 mg/ml caffeine

On further questioning, the patient admitted that he had recently been working extended hours as a taxi driver. In order to do this, he had been consuming one bottle (1000 ml) per week of a highly caffeinated (caffeine content 4.04 mg/ml) commercially available beverage for 6 months (Figure 2).

Six months after discontinuing the caffeine beverage, he was asymptomatic. A repeat echocardiogram confirmed that the left ventricular dimensions had returned to normal and the estimated ejection fraction was 65%.

## Comment

Caffeine is a methylxanthine, part of the biochemical family that includes theophylline and aminophylline. Methylxanthines are associated with cardiac and central nervous system stimulation, leading to their use in 'high energy' stimulant drinks. Chronic excess caffeine ingestion can result in tachycardia, arrhythmias and left ventricular dysfunction.

Caffeine's primary cardiac action is competitive antagonism of the adenosine cell surface receptor and has been shown to increase myocardial automaticity and potentiate the action of catecholamines [1]. Caffeine can alter calcium handling at an intracellular level by directly inhibiting calcium influx and leading to depressed left ventricular systolic and diastolic function [2]. Excessive caffeine intake may be a factor in the genesis of arrhythmias associated with caffeine toxicity [3]. Canine studies have suggested a dose-dependant arrythmogenicity of caffeine [4].

## Conclusion

The relationship between fast atrial fibrillation and reversible left ventricular failure was first described in 1949 by Phillips and Levine [5].

In this case report, chronic high dose caffeine ingestion has precipitated a tachycardia-induced cardiomyopathy, which fully resolved on discontinuation. The beverage consumed contained significantly greater levels of caffeine than tea (0.19 mg/ml) or instant coffee (0.52 mg/ml) [6]. This patient was drinking the equivalent of approximately 150 cups of tea or 55 cups of coffee per week in this stimulant drink alone.

## Competing interests

The author(s) declare that they have no competing interests.

## Authors' contributions

All authors (STCP, PM, SWD) were involved with the case and writing of the manuscript. All authors have read and approved the final manuscript.
